# Mobile tablet audiometry in fluctuating autoimmune ear disease

**DOI:** 10.1186/s40463-017-0195-1

**Published:** 2017-03-07

**Authors:** Scott Kohlert, Matthew Bromwich

**Affiliations:** 1 Faculty of Medicine, University of Ottawa, Ottawa, Canada; 2 Department of Otolaryngology – Head and Neck Surgery, The Ottawa Hospital, Ottawa, Canada; 3 Department of Otolaryngology – Head and Neck Surgery, Children’s Hospital of Eastern Ontario, 401 Smyth Rd, Ottawa, ON K1H 8 L1 Canada

**Keywords:** Audiometry, Tablet, Hearing loss, Audiology, Autoimmune inner ear disease

## Abstract

**Background:**

Autoimmune inner ear disease (AIED) is a rare condition characterized by bilateral fluctuating sensorineural hearing loss (SNHL). The labile nature of this hearing loss makes it difficult to accurately quantify with conventional methods, and therefore it is challenging to rehabilitate.

**Methods:**

Over a 9-month period one pediatric patient with severe AIED was monitored and conducted home audiograms using a previously validated testing system (Shoebox Audiometry). During this period he also underwent several clinical audiograms. The correlation between clinical and home audiograms was analyzed with a Pearson coefficient, and the range and frequency of fluctuations was recorded.

**Results:**

Sixty-four automated home audiograms and nine clinical audiograms were conducted. When tested at home using a calibrated system the pure tone average (PTA) fluctuated between 12 dB and 72 dB indicating large variability in hearing. Fluctuations were frequent: on 28 occasions the PTA varied by at least 5 dB when retested within 4 days. The mean PTA was 50 dB and 95% of the thresholds were within 36 dB of the mean. Clinical audiograms obtained on the same day or within 1 day of home testing were highly concordant (with a Pearson coefficient of 0.93).

**Conclusion:**

AIED can result in significant fluctuations in hearing over short periods of time. Home testing enables a more granular look at variations over time and correlates well with clinical testing, and thus facilitates rapid action and informed rehabilitation.

**Electronic supplementary material:**

The online version of this article (doi:10.1186/s40463-017-0195-1) contains supplementary material, which is available to authorized users.

## Background

Autoimmune inner ear disease (AIED) was first described in 1979 as a sensorineural hearing loss that is usually bilateral, often fluctuates, progresses over weeks to months and is responsive to treatments for autoimmune disease [1, 2]. It is a rare condition that is estimated to account for less than 1% of all hearing impairment [2]. While conventional sound booth audiograms are the gold standard for the assessment of a patient’s hearing, they are relatively time consuming, require the expertise of a trained audiologist, and provide only a brief snapshot of the patient’s hearing. While this does not pose a problem for most forms of hearing loss, it can be difficult to adequately assess and treat a patient with AIED using conventional audiometry alone given the rapidly fluctuating nature of this disease.

AIED can be classified as primary AIED (immune mediated disease limited to the inner ear) or secondary AIED (immune mediated inner ear disease as a manifestation of a systemic autoimmune process) [3]. Secondary AIED is associated with a wide array of systemic conditions including Behçet’s, Wegener’s, Hashimoto’s thyroiditis, rheumatoid arthritis, and lupus [2, 4, 5].

Corticosteroids are the gold standard medical treatment modality for AIED and have been shown to be beneficial in 70% of cases [6]. While corticosteroids are the only medications that have been consistently proven to be of benefit [7], several other therapies have been employed for the treatment of recalcitrant disease. Cyclophosphamide and methotrexate have been shown to reverse disease progression, although these are associated with significant toxicity including myelosuppression, infertility and increased risk of malignancy. Although costly and resource intensive, plasmapheresis has also been suggested as an adjunct to steroids or cytologic agents for patients with refractory disease [4], and has even been shown to allow as many as 75% of patients to wean from immunosuppression [8]. Biologic agents such as Etanercept and Rituximab have been studied with conflicting results [9, 10]. Studies have also investigated the role of commonly used transplant immunosuppressants such as azathioprine [11] and mycophenolate [12]. While showing promising results, these treatments are often associated with significant side-effect profiles and have not become commonly employed in the treatment of AIED.

As is the case with many types of hearing loss, rehabilitation with amplification can decrease morbidity. That said, amplification can be a challenge for patients with AIED given the rapidity and severity of fluctuations. Therapeutic planning (including steroids, hearing aids and implantation) should be guided by data, but currently only snapshots are available and no validated home diagnostics are available. Finally, patients who cannot tolerate or who have failed medical management can be considered for cochlear implantation. However, given the sometimes normal hearing of a person with AIED it is essential to frequently document the hearing troughs prior to proceeding with surgery in order to understand the patient’s real-world experience.

In order to create a clear picture of the rapid changes in hearing associated with AIED frequent audiograms are necessary. Typically, a routine paediatric audiogram takes 15 min at our institution, and is associated with an overall cost of approximately $300 CAD. Thus, it is not feasible to perform conventional audiometry on a daily basis. However, with the advent of mobile tablet audiometery (Shoebox Audiometer - Clearwater Clinical Limited, Ottawa, Canada) it is possible to obtain valid results in a quiet room using self-testing methods at home. The tablet audiometer is a calibrated iPad (Apple Inc, Cupertino California) application that is paired with standard audiometric transducers that enables patients to perform their own audiogram by playing a validated game. The tool is Health Canada approved as a medical device, and has been internally and externally validated as an accurate tool for self-assessing hearing outside of a conventional sound booth [13, 14].

Given the associated logistical and financial barriers associated with frequent audiometry, no previous study has used this technique to document the immense frequency and severity of fluctuations associated with AIED. Using the tablet audiometry device, our study aimed to use frequent home audiograms to evaluate the variability and progression of these fluctuations in a single pediatric (teenage) patient over a 9-month period.

## Methods

One patient with AIED, unilateral sensory-neural deafness and contralateral fluctuating hearing, was provided with an iPad audiometer and asked to perform home audiograms as frequently as possible to help him understand his own symptoms. Pure tone thresholds (from 500 to 8000 Hz) were collected using an automated tablet based system whereby users sort objects that either produce a calibrated sound or are silent in a forced alternate choice paradigm [13, 14]. Unmasked air conduction thresholds were performed. Each test was timestamped, and the results were encrypted and saved locally on the tablet. Throughout, the patient also underwent periodic conventional sound booth audiograms during his regularly scheduled clinical visits.

The range and frequency of fluctuations on the home audiograms were recorded. Pure tone averages were calculated for each audiogram. The mean, median, minimum, and maximum values were determined. The correlation between clinical and home audiograms was analyzed using a Pearson’s Correlation Coefficient.

Research ethics approval for this project was sought from the Children’s Hospital of Eastern Ontario Research Ethics Board, and an exemption was obtained. Written consent was obtained from the patient permitting collection and publication of his clinical history and audiometry data for research purposes.

## Results

Sixty-four automated home audiograms and nine routine clinical audiograms were conducted over a 9-month period using the tablet audiometer device. Home audiograms were performed within 4 days of the previous test in 58/64 (91%), and 57/64 (89%) were performed in the first 3 months of the study. The patient was highly compliant with home audiometry in the first 3 months, performing one test every 1.6 days. He became less compliant in the subsequent 6 months, performing testing only once every 25.9 days.

**Fig. 1:**
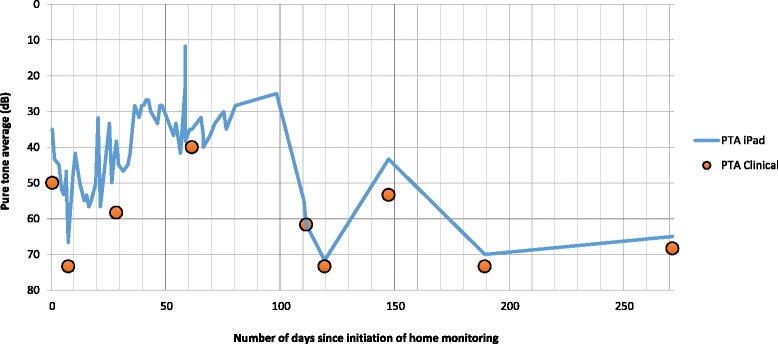
Fluctuations in Pure Tone Average (PTA) over time

**Fig. 2:**
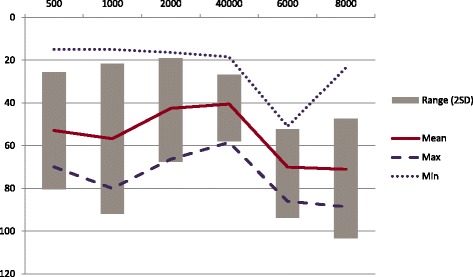
Range of Pure Tone Thresholds - Min, Max, Mean and Range (2SD)

The pure tone average (PTA) calculated with the home audiometer fluctuated frequently. On 28 occasions the PTA varied by at least 5 dB on a subsequent test occurring within 4 days of the previous (Table 1), and on 6 occasions the PTA fluctuated by more than 15 dB within 24 h. 5 dB was chosen as the limit for significance, as this is widely felt to be the test-retest threshold for clinical pure-tone audiometry [15]. The patient’s PTA fluctuated between 12 dB and 67 dB in the 3-month span where he was highly compliant with testing (and between 12 dB and 72 dB over the entire length of the study), indicating a large variability in hearing. The labile nature of the patient’s hearing is further highlighted in Fig. 1. The mean PTA was 50 dB and 95% of the thresholds were within 36 dB of the mean (Fig. 2).

**Table 1:** Home audiograms performed within 4 days of previous where PTA fluctuated by at least 5 dB when compared to previous

Test number	Days from prior test	Fluctuation (dB)
2	1	8
4	1	7
6	1	5
8	1	20
10	2	18
11	1	7
12	2	8
13	2	5
17	2	5
18	1	18
19	1	25
20	4	23
21	1	17
22	1	7
23	1	5
24	1	7
28	1	7
29	1	7
38	1	5
41	3	5
43	2	8
44	2	18
45	0	12
46	0	27
51	1	5
56	1	5
57	4	7
60	1	7

Clinical audiograms obtained on the same day or within 1 day of home testing were highly concordant, with a Pearson coefficient (R) of 0.93 (Fig. 1). A quick glance at Fig. 1 shows seemingly more variation in the results early in the study, however this is likely due to the more frequent nature of the audiograms at that time.

## Discussion

AIED is a rare and relatively poorly understood condition that can lead to profound hearing loss and significant patient morbidity [2, 4–6, 16]. It is seemingly more prevalent in women, and most commonly presents in the third to sixth decade of life [2]. While patients will often present with unilateral hearing loss, approximately 80% patients will eventually develop bilateral disease [2, 6] (although the severity of the loss is often asymmetric [7, 17, 18]). Up to 50–80% of patients will develop associated otologic symptoms including tinnitus and aural fullness [4, 6] and the incidence of associated vestibular symptoms is as high as 79% in the literature [6]. These associated symptoms will often fluctuate along with the hearing [4]. The hearing loss and associated symptoms often worsen progressively over a period of weeks to months, although sudden deafness or severe vestibular hypofunction has been reported [19]. The prognosis is usually poor with progression to severe losses by the age of 30.

The patient described herein reported fluctuating hearing at home, however it was unclear to the treating team the range and rapidity of the variability. This presented as difficulty using standard amplification and problems with school participation. Our study has demonstrated the highly fluctuating nature of AIED, and has shown that regular home audiograms can be a feasible and reliable way to monitor a patient’s clinical progression over time. In this patient’s case standard intratympanic steroid therapy was ineffective over several months. We were initially hesitant to proceed with a cochlear implant given that the patient still had hearing in one ear. However, we were unaware of the severity and frequency of their problems until daily monitoring was undertaken. As a result they received the implant in the contralateral ear much sooner than would otherwise occurred, with the knowledge that some days the patient will have two functional ears and other days not.

We propose that clinicians could use home audiometry devices for a wide array of uses in the AIED patient population. Tablet audiometry can be used to document patients’ progression over time, identify major fluctuations earlier in the disease process, as well as monitor their response to treatment. The benefit of corticosteroid therapy for AIED has been shown to wane over time [7], and earlier detection of recalcitrant hearing loss may allow specialists to initiate advanced treatment sooner. Data-driven therapy has the potential to decrease not only the patient morbidity associated with worsening hearing loss, but that associated with treatment side effects. Corticosteroids are associated with a long list of adverse side effects including weight gain, diabetes, immunosuppression, osteoporosis and avascular necrosis of the femoral head. By using regular audiogram data to identify the point where corticosteroid therapy has outlived its clinical benefit, physicians can discontinue therapy as soon as it becomes ineffective, reducing the risk of adverse events associated with the medication.

Access to home audiometry may assist patients with their rehabilitation as patients with fluctuating losses complain of limited utility of hearing aids due to the constantly changing fit. Having access to daily home audiometry results may enable patients to more accurately refit their hearing aids in response to fluctuations in their hearing throughout the week. Already, many modern hearing aids allow patients to manually select programs on their hearing aids using an app on their mobile phone. Further, new models for the sale and fitting of hearing aids are emerging with the advent of ‘Self-fit hearing aids over-the-Internet’ and ‘Personal Sound Amplification Products’ (PSAPs). Indeed, there is a new trend in medicine of increased patient generated data and patient ownership of that data. In order to ensure reliable results and responsible care of the patient the medical community must participate in this evolution or risk being supplanted. However, several barriers to this method due exist, including access to appropriate home equipment, access to suitably quiet environments and the level of training required to administer and interpret results. Previous research has demonstrated that un-calibrated headphones or ear-buds do not provide reliable results and that there is great variability between different types of home hearing tests in terms of accuracy and ability to handle background noise, however, calibrated systems can perform well in suitable environments. [20–22]. There is also still debate about who is appropriate to be tested at home and who is capable of performing the test. Nevertheless, with the burgeoning of on-line training, gamified testing and artificial intelligence diagnostic support we see a path where future research and development could greatly improve the efficacy of testing and rehabilitation in this challenging population.

While our study only investigated one patient with AIED, further work could be done to collect regular audiometry data from a larger cohort of patients with AIED or other conditions such as Meniere’s disease or examine the speed of patient hearing recovery from surgery. Patient generated data appears to be useful, informative and satisfying to patients in other areas of medicine and this may be similar.

## Conclusion

AIED can result in significant fluctuations in hearing over short periods of time. This study is the first of its kind to prospectively monitor a pediatric patient suffering from AIED using frequent home audiograms. We have demonstrated that frequent calibrated home- audiometry is a viable and accurate method of measuring the frequency and severity of fluctuations in hearing over time in patients with AIED. Home testing enables a more granular look at variations over time and correlates well with clinical testing, and thus facilitates informed rehabilitation. Clinicians can employ home audiometry to accurately document disease progression, monitor response to treatment, and potentially improve patient satisfaction by improving the utility of amplification devices.

## Additional file

Additional file 1: Complete audiometric dataset. (XLSX 31 kb)

## Abbreviations

AIED: Autoimmune inner ear disease
CAD: Canadian Dollars
dB: Decibels
PSAPs: Personal sound amplification products
PTA: Pure tone average
R: Pearson coefficient
